# A cross-sectional observational study on allergen-specific IgE positivity in a southeast coastal versus a southwest inland region of China

**DOI:** 10.1038/s41598-017-10109-3

**Published:** 2017-08-30

**Authors:** Guangqiao Zeng, Wenting Luo, Zehong Wu, Ling Li, Peiyan Zheng, Huimin Huang, Nili Wei, Jiaying Luo, Baoqing Sun, Yong Liu

**Affiliations:** 1 State Key Laboratory of Respiratory Disease, Guangzhou, 510120 Guangdong, China; 2 National Clinical Research Center of Respiratory Disease, Guangzhou, 510120 Guangdong, China; 3 Guangzhou Institute of Respiratory Disease, Guangzhou, 510120 Guangdong, China; 4 Department of Allergy and Clinical Immunology, First Affiliated Hospital of Guangzhou Medical University, Guangzhou, 510120 Guangdong, China; 5 Sino-French Hoffmann Institute, Guangzhou Medical University, Guangzhou, 510120 Guangdong, China; 6 Guangzhou Kingmed Diagnostics Group Co., Ltd., Guangzhou, 510120 Guangdong, China

## Abstract

Few studies addressed trans-regional differences in allergen sensitization between areas within a similar latitudinal range but with distinct geomorphological features. We investigated specific IgE (sIgE) positivity to common allergens in populations from two southern China provinces. Using a uniformed protocol, serum samples were collected from 2778 subjects with suspected atopy in coastal Guangdong and inland Yunnan. The overall prevalence of sIgE positivity were 57.8% (95% CI: 56.0%, 59.6%) from Guangdong vs 60.9% (95% CI: 59.1%, 62.7%) from Yunnan. House dust mite (d1) was the most common allergen in both regions. Among d1-sensitized subjects, only 35.7% (208/583) in Guangdong and 22.9% (147/642) in Yunnan tested positive for d1 alone. Among those poly-sensitized d1-positive subjects, cockroach was the most common co-sensitizing aeroallergen. 41.9% of the d1-sensitized Guangdong subjects showed high-class sIgE reactivity (≥class 4), in contrast to a very low percentage of such reactivity in Yunnan. However, 36.3% of d1-sensitized subjects in Yunnan were concomitantly positive for tree pollen mix. Surprisingly, Yunnan subjects showed high prevalence of sIgE positivity for crabs and shrimps, either by overall or by age-group analysis, compared with their Guangdong counterparts (both *P* < 0.05). These findings may add to data about local allergies in China and worldwide.

## Introduction

Studies have shown that allergens as a cause of allergic diseases exhibit regional characteristics in distribution given their broad spectrum. For example, whereas grass pollens are the most common inhaled allergens in Europe, USA and Central Africa^1–4^, house dust mites are the major aeroallergens in Asian countries^5^. In Israel, patients with allergic rhinitis are dominated by dust mite-sensitization in the humid coastal areas and by fungus-sensitization in the dry desert cities^6^. Conceivably, in a vast territory like China, trans-regional difference in allergens may be conspicuous and would represent an important clue to evidence-based prevention and treatment of local allergies. During the past decades, the prospering economy, increasing urbanization, changing lifestyle and climatic factors in this country have further complicated the geographical variations in allergen distribution.

**Figure 1:**
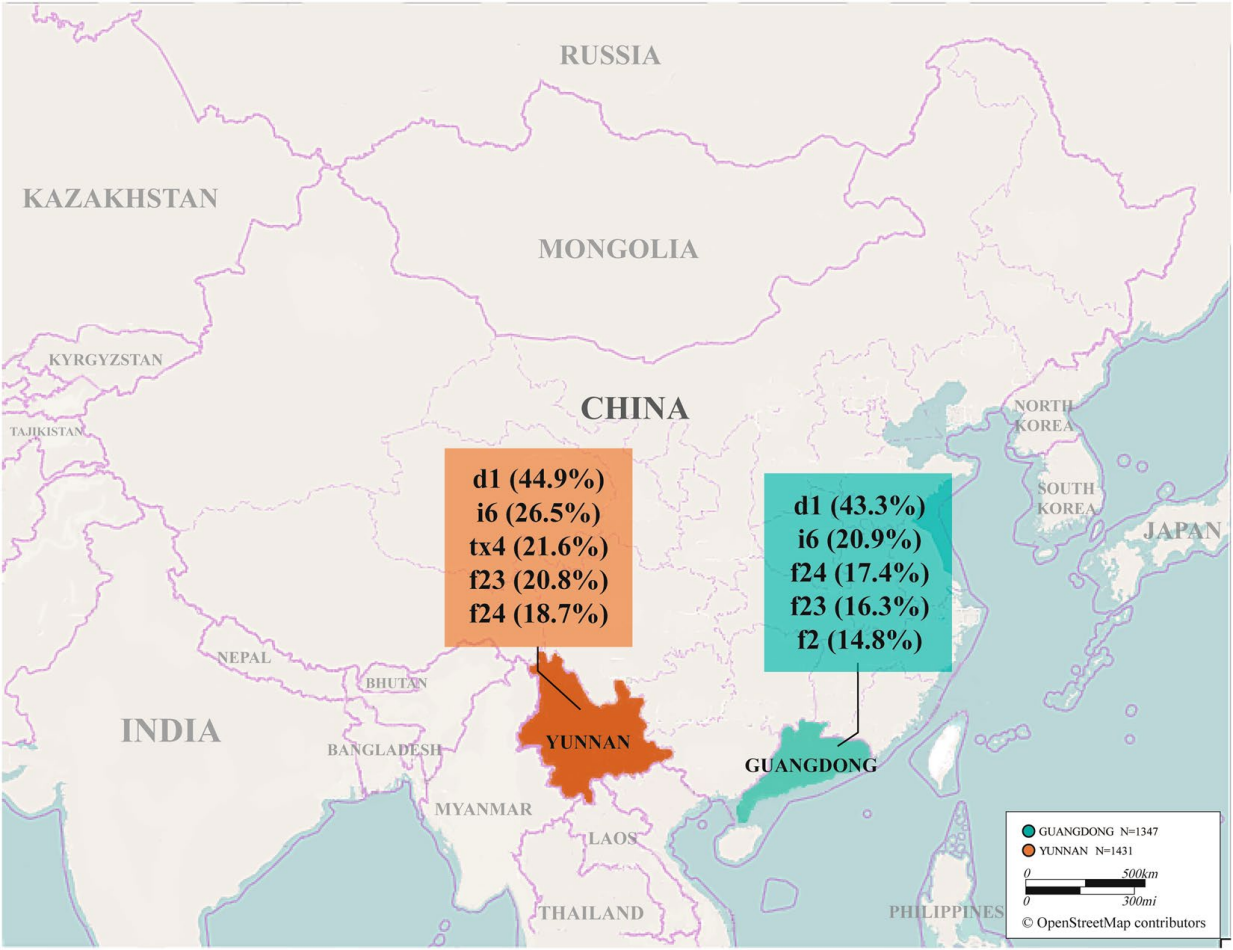
Sensitization to individual allergens in Guangdong and Yunnan. The first five leading allergens in Guangdong were d1 (43.3%, 95% CI: 41.4%, 45.1%), i6 (20.9%, 95% CI: 19.4%, 22.4%), f24 (17.4%, 95% CI: 14.3%, 20.5%), f23 (16.3%, 95% CI: 14.9%, 17.7%), and f2 (14.8%, 95% CI: 13.5%, 16.1%), compared with d1 (44.9%, 95% CI: 43.0%, 46.7%), i6 (26.5%, 95% CI: 24.8%, 281%), tx4 (21.6%, 95% CI: 18.2%, 25.0%), f24 (20.8%, 95% CI: 17.5%, 24.1%), and f23 (18.7%, 95% CI: 15.5%, 21.9%) in Yunnan. The figure was generated from OpenStreepMap® (http://www.openstreetmap.org/), modified and annotated with Adobe Illustrator CS6 (Adobe, San Jose, California, USA). The cartography in this figure is copyrighted by © OpenStreetMap contributors, and licensed under the Creative Commons Attribution-ShareAlike 2.0 license (CC BY-SA). For permission to use this map from OpenStreepMap®, please refer to: http://www.openstreetmap.org/copyright/en. d1: house dust mite; i6: cockroach; tx4: tree pollen mix; f23: crab; f24: shrimp; f2: milk.

Guangdong is a southeastern province of the Mainland China facing the South China Sea, located within latitudes 20°09′N–25°31′N and longitudes 109°45′E–117°20′E, with a total population of 108 million (as of 2015). In this coastal region, the landforms chiefly consist of hills and plains, and the climate is characteristic of tropical and subtropical monsoon marine zone, i.e. with a mean temperature ranging between 18 °C and 24 °C almost of the year, and an abundant rainfall in the summer. To the west of Guangdong at a straight-line distance of 840 miles away, there stretches Yunnan, completely an inland province located on a low-latitude plateau in the southwestern China, bordering other Chinese provinces in the east and north, Myanmar in the west, and Laos and Vietnam in the south, and with a total population of 47.4 million (as of 2015). Interestingly, although located within the similar latitudes range (21°09′N–29°15′N) as is Guangdong, Yunnan province covers diverse climatic zones (tropical, temperate, and frigid), with vertical and horizontal changes in perennial temperature and rainfall owing to the influence of landforms, elevation, and latitudes. Overall, Guangdong and Yunnan provinces belong to typical parts of the southern coastal and inland regions in China, respectively, showing obvious dissimilarity in terms of natural environments between each other (Fig. 1).

There have been several studies^7–10^ reporting separately on the prevalence of allergen sensitization in Guangdong and Yunnan provinces of China. However, there are currently no published studies comparing these data between the two regions using a uniform protocol of allergen detection approach. Even if from a global perspective, large-sample studies have rarely been conducted to investigate allergen sensitization across regions of similar latitudinal distribution and meanwhile distinct geological climates within a single country.

To the latest of our knowledge, the present study may be the first one to detect serum-specific immunoglobulin E (sIgE) levels in the southeast coastal and southwest inland areas of China based on a standardized allergen detection method. We intended to examine the differences in sensitization to local allergens in subjects with suspected atopy between Guangdong and Yunnan. We speculated that such study attempt would help add to evidence that supports diagnosis and treatment of local allergies specific to geographic regions.

## Methodology

### Ethics consideration

This study was approved by the Institutional Review Board of First Affiliated Hospital (GYFYY-2013-02-19) of Guangzhou Medical University, and registered in the Chinese Clinical Trial Registry (registration number: ChiCTR-DCC-13004003). Written informed consent was obtained from all participants. All methods, including the study implementation and laboratory investigation, were performed in accordance with Declaration of Helsinki, Committee on Publication Ethics (COPE) guidelines, and China legislation on use of human serum samples for research purposes. The subjects and data reported by this study, all or in part, have not been included in any previous publications.

### Study design and population

This was a cross-sectional observational study involving serum samples from the two provinces, conducted between January 2014 and December 2015. In Guangdong province, techniques for allergen-specific sIgE measurements using full-automatic detectors have been increasingly mature over the past years in supporting laboratory diagnostics for local allergies. Currently, our Allergy Information Repository of State Key Laboratory of Respiratory Disease (AIR-SKLRD) in Guangzhou, the capital metropolis of Guangdong, keeps in storage a huge bio-bank of serum samples from informed consenting subjects throughout the entire province who were referred to us for allergy evaluation. Thus, the AIR has become the largest dataset of allergic patient information in southern China. However, in the economically less-developed Yunnan, sophisticated laboratory investigations for allergy diagnosis remain inadequately available or accessible to allergists. To ensure the uniform standard of testing for this trans-regional study, AIR-SKLRD collaborated with a certified third-party laboratory services provider, the Kingmed Diagnostics (KMD at http://en.kingmed.com.cn/, with accreditation by ISO/IEC17025; ISO9001; ISO15189; and recognition by College of American Pathologists; American National Glycohemoglobin Standardization Program), in developing a mutually agreed implementation protocol. As per this protocol: (1) KMD was exclusively responsible for allergen-specific sIgE testing of all serum samples in the present study, and submitting the resultant data to AIR-SKLRD for final analysis. (2) Collection, preparation and transportation of serum samples of Guangdong subjects were completed by AIR-SKLRD, and those of Yunnan subjects, by a designated local team of KMD branch. All procedures were completed with strict adherence to the protocol. (3) Prior to study, KMD held a two-day workshop on standard requirements for sample delivery and processing to all investigators; in turn, the trained staff and investigators maintained an effective communication with local health professionals who referred their patients for the tests.

**Figure 2:**
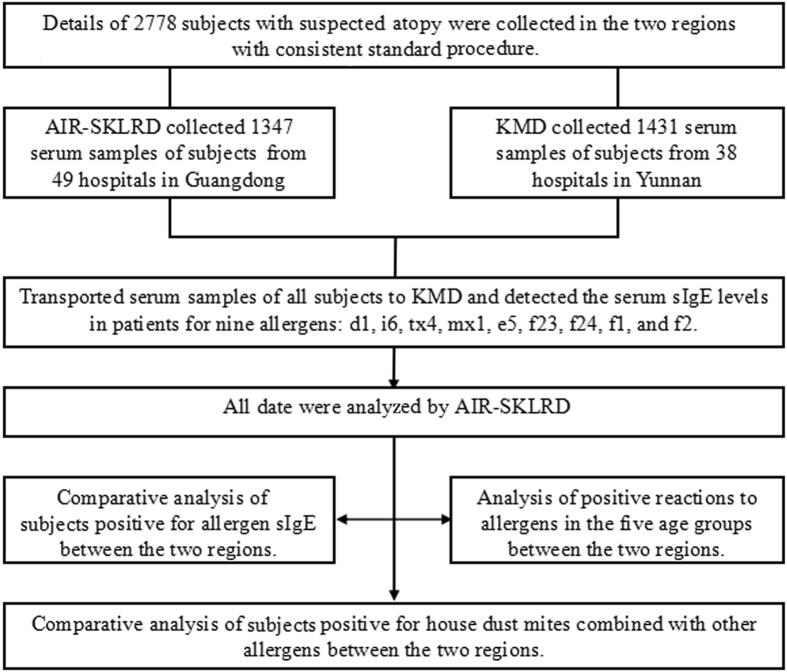
The flow-chart of the present study.

Our sampling strategy focused on a convenience sample of referrals for allergen sIgE testing from secondary and tertiary hospitals in the two provinces within a two-year duration. Compared with a strictly random one, a convenience sample of hospital-referred sera could be practically more feasible. For inclusion criteria, atopic individuals tested for serum sIgE had to present with clinical signs of suspected allergies, defined as at least one of the following: symptoms of skin allergy (such as wheal, rashes, or urticaria), asthma (such as wheezing, dyspnea and/or cough not attributable to common cold), allergic rhinitis (such as sneezing, runny nose, nasal obstruction/itching), or a positive skin prick test. To control for conditions that may interfere with serum sIgE levels, samples from those on specific immunotherapy, with immunodeficiency or parasitic infection, were excluded. During the study period, KMD received 2778 eligible serum samples collected from 1431 subjects referred by 38 hospitals (13 secondary and 25 tertiary institutions) in Yunnan, and from 1347 subjects by 49 hospitals (18 secondary and 31 tertiary institutions) in Guangdong. All referring hospitals were evenly distributed across these two provinces. Among all contributors of serum samples, the 680 (47.5%) from Yunnan and 656 (48.7%) from Guangdong were males; 725 (50.6%) and 679 (50.4%), respectively, lived in an urban environment. There were no difference in gender and urban/rural residence between the two provinces. However, serum contributors from Guangdong were younger than those from Yunnan [median age: 11 years (range 1–86) vs 23 years (range 1–89)]. The flow chart of study is shown in Fig. 2.

### Laboratory techniques

#### Sample collection, processing and storage

Five milliliters of venous blood was collected from all subjects with separation gel containing vacutainer tubes. The samples were then centrifuged at 3000 rpm for 10 min. The supernatant was recovered for use in subsequent detections. The test specimens were delivered via dry-ice cold chain logistics to the standardized ImmunoCAP laboratory of KMD. During the study, repeated freezing and thawing of the sera were avoided by storing the remaining sample in a −80 °C freezer.

#### Allergen-specific IgE detection

A full-automated *in vitro* allergen detector, ImmunoCAP 1000 system (Thermo Fisher Scientific Inc., California, USA), was used to detect allergen-specific sIgE in the sera, as described elsewhere^11, 12^. According to previous surveys in southern China, house dust mites, cockroaches, dog dander, and molds are the most common aeroallergens^7, 12, 13^. Eggs and milk are the major food allergens affecting children^8, 13, 14^. Shrimps and crabs are the important food allergens sensitizing adults living in coastal regions^13^. Moreover, the warm and humid climate of southern China favors rampant growth of trees. Therefore, sIgE to nine categories of allergens (ImmunoCAP number, species source) was evaluated in this study, including house dust mite (d1: house dust mite, *Dermatophagoides pteronyssinus*), cockroach (i6, *Blatella germanica*), tree pollen mix (tx4: *Quercus alba*, *Ulmus americana*, *Platanus acerifolia*, *Salix caprea*, *Populus deltoides*), mold mix (mx1: *Penicillium chrysogenum*, *Cladosporium herbarum*, *Aspergillus fumigatus*, *Alternaria alternata*), dog dander (e5), crab (f23), shrimp (f24), egg white (f1), and milk (f2).

Positive reactivity was defined as an sIgE level ≥0.35kU/L (class 1 or above). According to the absolute sIgE levels, the reactivity was categorized quantitatively into six classes: Class 1 (≥0.35 to <0.70 kUA/L), Class 2 (≥0.70 to <3.50 kUA/L), Class 3 (≥3.50 to <17.50 kUA/L), Class 4 (≥17.50 to <50.00 kUA/L), Class 5 (≥50.00 to <100.00 kUA/L), and Class 6 (≥100.00 kUA/L)^15, 16^.

### Statistical analysis

Data were processed and analyzed with IBM SPSS Statistics for Windows Version 22.0 (IBM Corp, Armonk, NY, USA). The prevalence of sIgE positivity was presented as percentage and 95% confidence intervals, and compared between Guangdong and Yunnan provinces with adjustment for age and/or gender when applicable. Chi-square test or Fisher’s exact probability method was used to determine the between-group differences in prevalence of sIgE positivity according to different regions, gender, allergens and age groups. The Bonferroni correction was performed to correct *P* values of multiple pairwise comparisons. Radar charts were used to compare different characteristics of single or multiple subjects. *P*-values below 0.05 were considered statistically significant.

## Results

### Overall and age-specific sensitization to any allergen

The prevalence of any sIgE positivity in the study population were 57.8% (95% CI: 56.0%, 59.6%) for Guangdong and 60.9% (95% CI: 59.1%, 62.7%) for Yunnan, respectively, when adjusted for age. Of those who test positive for any sIgE, 69.3% were sensitized to ≥2 allergens in Yunnan, compared to a lower percentage (56.5%) of their Guangdong counterparts (*X* ^2^ = 29.540, *P* < 0.01). In either province, male subjects had significantly higher overall prevalence of sensitization than females (*X* ^2^ = 14.751, *P* < 0.001 *X* ^2^ = 9.323, *P* < 0.05). We stratified the study population by age to account for the subjects in different life stages as described in previous studies^13, 17^: infancy (years 0–3): 291 in Guangdong and 186 in Yunnan), preschool age (years 4–6: 223 in Guangdong and 170 in Yunnan), older children (years 7–14: 224 in Guangdong and 293 in Yunnan), young adulthood (years 15–50: 549 in Guangdong and 629 in Yunnan) and late adulthood (years 51 and above: 60 in Guangdong and 153 in Yunnan). When stratified by age, allergen sensitization showed interesting differences among age groups in both Guangdong and Yunnan (*X* ^2^ = 85.824, *P* < 0.001; *X* ^2^ = 10.401, *P* < 0.05). In Guangdong, the overall prevalence of allergen sensitization was relatively high among pediatric subjects (0–14 years of age), and was the highest in age group 7–14 years (75.3%, 95% CI: 69.2%, 81.3%). By contrast, the data from Yunnan were high for all ages. Specifically, Yunnan subjects aged 15–50 years had the highest prevalence of sIgE positivity (63.3%, 95% CI: 59.9%, 66.8%), which was significantly higher compared with subjects of the same age group in Guangdong (47.3%, 95% CI: 43.3%, 51.4%; *X* ^2^ = 33.805, *P* < 0.001). In both provinces, the age group ≥51 years showed the lowest prevalence of any sIgE positivity (Table 1).

**Table 1:** Overall and stratified prevalence of positive sIgE to any allergen in the study population (n = 2778).

	Prevalence of sIgE positivity, % (95% CI)	
	Guangdong	Yunnan
^a^Overall prevalence of positivity	57.8% (56.0%, 59.6%)	60.9% (59.1%, 62.7%)
Prevalence of sIgE positivity by *sensitizing allergens*		
1	43.6% (40.1%, 47.0%)	30.7% (29.6%, 35.9%)
2	18.7% (16.0,%, 21.4%)	22.9% (20.1%, 25.7%)
3	11.1% (8.9%, 13.2%)	16.8% (14.4%, 19.3%)
4	16.1% (13.6%, 18.7%)	16.7% (14.2%, 19.2%)
5	5.8% (4.2%, 7.5%)	6.4% (4.8%, 8.0%)
≥6	4.7% (3.2%, 6.2%)	6.4% (4.8%, 8.0%)
*Gender*		
Male	63.7% (460.0%, 67.4%)^**^	65.5% (61.8%, 69.2%)^**^
Female	53.4% (49.7%, 57.1%)	57.5% (54.1%, 61.0%)
^b^ *Age*		
0–3 years	67.7% (62.3%, 73.1%)	56.6% (48.3%, 64.9%)
4–6 years	67.7% (61.6%, 73.8%)	62.7% (53.7%, 71.8%)
7–14 years	75.3% (69.2%, 81.3%)	62.1% (56.6%, 67.7%)
15–50 years	47.3% (43.3%, 51.4%)	63.3% (59.9%, 66.8%)
≥51 years	33.3% (21.4%, 45.3%)	50.3% (42.4%, 58.2%)

Data were presented as proportion and 95% confidence interval (95% CI). ^a^There was no statistical difference in overall prevalence of sIgE positivity between Guangdong (57.8%, 95% CI: 56.0%, 59.6%) and Yunnan (60.9%, 95% CI: 59.1%, 62.7%) when adjusted for age (*X* ^*2*^ = 3.719, *P* > 0.05). ^**^ *P* < 0.001; The prevalence of sIgE positivity was significantly higher in male than in female subjects in either province (Guangdong: *P* < 0.001; Yunnan: *P* < 0.05). Allergen sensitization showed significant differences among age groups in both Guangdong (*P* < 0.001) and Yunnan (*P* < 0.05), ^b^The Bonferroni correction was used to correct *P* values of pairwise comparison among five age groups.

### Overall sensitization to individual allergens

**Figure 3:**
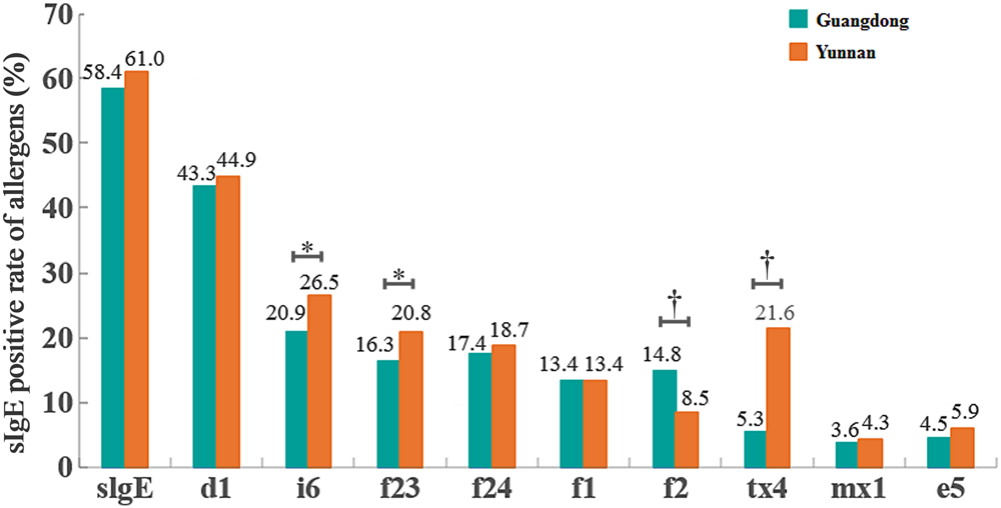
Detection of nine allergens in subjects with suspected atopy in Guangdong and Yunnan. *P < 0.05, ^†^ *P* < 0.001. The prevalence of sIgE sensitization for i6, tx4, and f23 in Yunnan was significantly higher than those in Guangdong (all *P* < 0.01), whereas the opposite was true for the f2-sIgE positivity (*P* < 0.001). d1: house dust mite; i6: cockroach; tx4: tree pollen mix; mx1: mold mix; e5: dog dander; f23: crab; f24: shrimp; f1: egg white; f2: milk.

**Figure 4:**
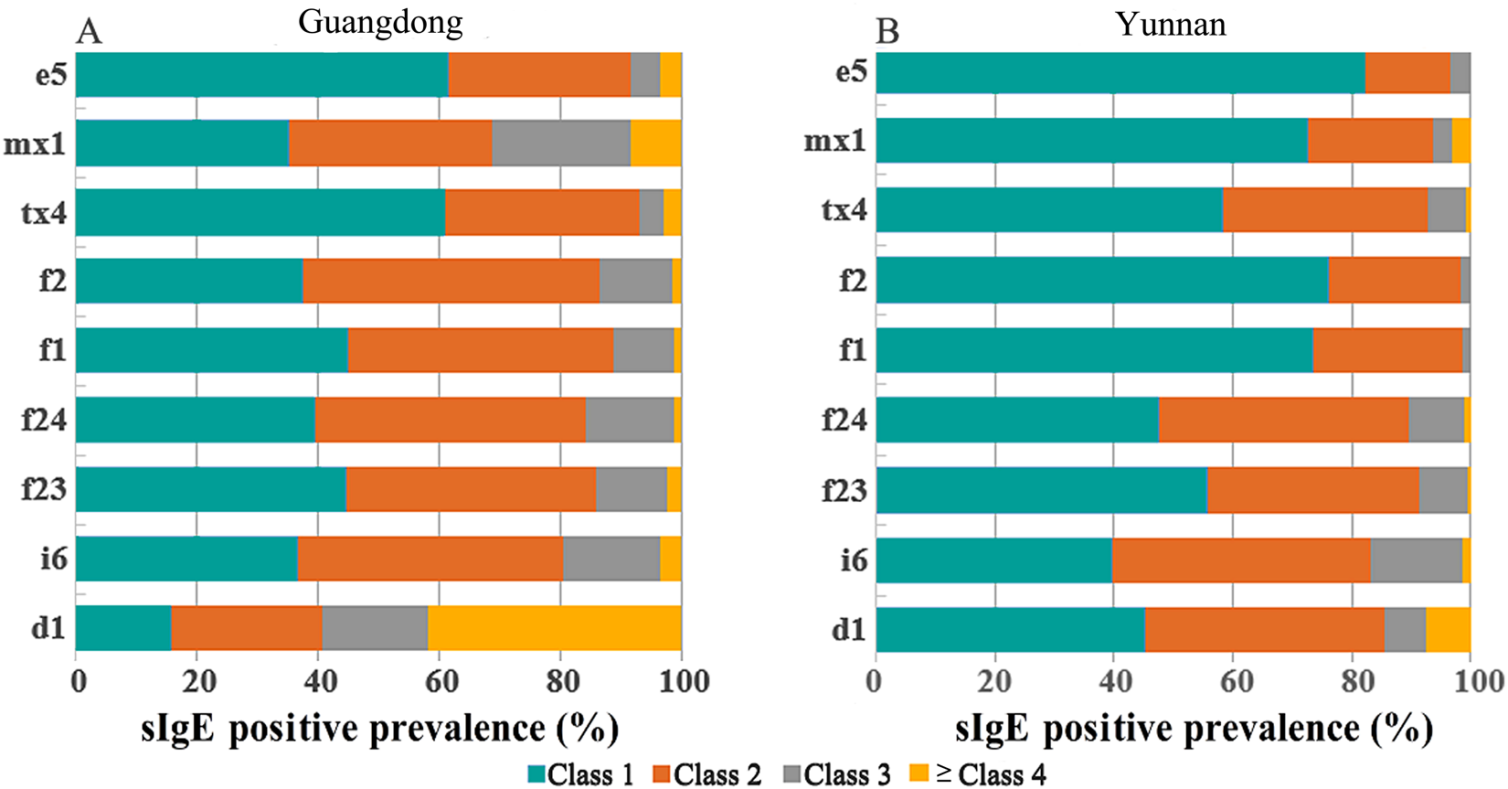
Classification of sIgE positive reactivity for individual allergens in Guangdong (**A**) and Yunnan (**B**) (%). Regarding the severity for individual allergens in both regions, the majority of allergens caused low-level sIgE reactivity (Classes 1–2) except for d1 which caused high-class reactivity (≥class 4 or above) in 41.9% of d1-positive Guangdong subjects. d1: house dust mite; i6: cockroach; tx4: tree pollen mix; mx1: mold mix; e5: dog dander; f23: crab; f24: shrimp; f1: egg white; f2: milk.

By increasing prevalence of sensitization to individual allergens, the top five major allergens were similar between the two regions, being house dust mite, cockroaches, shrimp, crab, and milk in Guangdong vs house dust mite, cockroaches, tree pollen mix, shrimp, and crab in Yunnan (Fig. 1). Guangdong subjects were more likely to have positive sIgE reactivity to milk compared with those from Yunnan (*X* ^2^ = 26.505, *P* < 0.001). Sensitization to tree pollens (tree pollen mix) was obviously more prevalent in Yunnan, which was nearly 4 times as found in Guangdong [21.6% (95% CI: 20.1%, 23.2%) vs 5.3% (95% CI: 4.5%, 6.1%), *X* ^2^ = 153.804, *P* < 0.001]. Surprisingly, there was also a higher prevalence of sIgE positivity for crab in the inland Yunnan than in the coastal Guangdong province of China [20.8% (95% CI: 17.5%, 24.1%) vs 16.3% (95% CI: 14.9%, 17.8%), *X* ^2^ = 9.275, *P* < 0.01] (Fig. 3). By the severity of individual sIgE reactivity, sensitization to almost all allergens were usually mild (class 2 or below) in subjects from either region. But importantly, while the both provinces showed comparable prevalence of house dust mite (d1) reactivity, 41.9% of the sensitized Guangdong subjects showed high-class reactivity (≥17.5 kU/L or Class 4) in drastic contrast to a very low percentage of such reactivity in Yunnan subjects (Fig. 4).

### Age-specific sensitization to individual allergens

Age-specific sensitization to individual allergens in Guangdong and Yunnan was investigated (Table 2). Among food allergens, egg white and milk were more likely to affect young children in either region, with the prevalence of sIgE positivity being highest before age 3, which were significantly higher than other age groups (all *P* < 0.001), and declining with older age. There were significant differences in prevalence of positivity for crab-sIgE and shrimp-sIgE across age groups in Guangdong (*X* ^2^ = 56.004, *X* ^2^ = 44.941, both *P* < 0.001), but in Yunnan, only the prevalence of crab-sIgE positivity was statistically different across age groups (*X* ^2^ = 10.849, *P* < 0.05). In both regions, the prevalence of crab-sIgE or shrimp-sIgE positivity was high in adults, with a peak at 15–50 years. Among aeroallergens, the prevalence of sIgE positivity for house dust mite showed significant age-group difference only in Guangdong (*X* ^2^ = 104.121, *P* < 0.001), with an increasing trend between 0–14 years, and peaking at 7–14 years (67.5%). In Yunnan, children aged 4–6 years appeared to have the highest prevalence of house dust mite sensitization (49.1%), although the age-group difference did not reach the level of statistical significance. The prevalence of sIgE positivity for cockroaches significantly varied with age groups in both regions (*X* ^2^ = 52.970, *P* < 0.001 in Guangdong; *X* ^*2*^ = 18.077, *P* < 0.01 in Yunnan), showing higher prevalence along with older age until peaking at 15–50 years and 7–14 years, respectively.

**Table 2:** Prevalence of allergen sIgE-positivity among age groups in Guangdong and Yunnan.

	Inhalant allergens % (95% CI)					Food allergens % (95% CI)			
	d1	i6	tx4	mx1	e5	f1	f2	f23	f24
Guangdong									
0–3 years	28.2 (23.0, 33.3)	7.2 (4.2, 10.2)	4.5 (2.1, 6.8)	1.4 (0.0, 2.7)	1.0 (−0.1, 2.2)	37.1 (31.6, 42.7)	43.0 (37.3, 48.6)	4.8 (2.4, 7.3)	7.6 (4.5, 10.6)
4–6 years	57.8 (51.4, 64.3)	20.2 (14.9, 25.4)	6.7 (3.4, 10.0)	6.7 (3.4, 10.0)	4.0 (1.5, 6.6)	20.6 (15.3, 25.9)	21.1 (15.7, 26.4)	13.5 (9.0, 17.9)	13.9 (9.4, 18.4)
7–14 years	67.5 (60.9, 74.1)	22.2 (16.3, 28.0)	3.6 (1.0, 6.2)	4.6 (1.7, 7.6)	8.8 (4.8, 12.7)	9.3 (5.2, 13.4)	10.8 (6.5, 15.2)	14.9 (9.9, 20.0)	14.9 (9.9, 20.0)
15–50 years	38.9 (34.9, 42.8)	28.2 (24.5, 24.0)	6.0 (4.1, 8.0)	3.1 (1.7, 4.5)	4.8 (3.1, 6.6)	1.0 (−1.2, 7.9)	0.9 (0.1, 1.6)	24.0 (20.5, 27.5)	24.9 (21.3, 28.4)
≥51 years	26.7 (15.5, 37.9)	15.0 (6.0, 24.0)	3.3 (−1.2, 7.9)	3.3 (−1.3, 7.9)	5.0 (−0.5, 10.5)	2 (3.3)	1.7 (−1.6, 4.9)	11.7 (33.5, 19.8)	15.0 (6.0, 24.0)
^a^ *P* value	<0.001	<0.001	>0.05	<0.05	<0.01	<0.001	<0.001	<0.001	<0.001
Yunnan									
0–3 years	38.2 (30.1, 46.4)	16.2 (10.0, 22.4)	21.3 (14.4, 28.2)	10.3 (5.2, 15.4)	15.4 (9.4, 21.5)	30.9 (23.1, 38.6)	34.6 (26.6, 42.6)	14.7 (8.8, 20.7)	13.2 (7.5, 18.9)
4–6 years	49.1 (39.7, 58.4)	18.2 (11.0, 22.4)	23.6 (15.7, 31.6)	4.5 (0.7, 8.4)	5.5 (1.2, 9.7)	21.8 (14.1, 29.5)	13.6 (7.2, 20.0)	13.6 (7.2, 20.0)	10.9 (5.1, 16.7)
7–14 years	46.4 (40.7, 52.1)	30.4 (25.1, 35.6)	21.8 (17.1, 26.6)	4.1 (1.8, 6.4)	6.1 (3.4, 8.9)	11.3 (7.6, 14.9)	7.2 (4.2, 10.1)	21.5 (16.8, 26.2)	18.4 (14.0, 22.9)
15–50 years	46.3 (42.7, 49.9)	29.1 (25.8, 32.4)	22.2 (19.2, 25.2)	3.5 (2.2, 4.8)	4.7 (3.2, 8.9)	10.7 (8.5, 12.9)	4.6 (3.1, 6.1)	22.7 (19.7, 25.8)	21.4 (18.4, 24.3)
≥51years	37.9 (30.2, 45.6)	21.6 (15.1,28.1)	16.3 (10.5, 22.2)	3.3 (0.5, 6.1)	3.3 (0.5, 6.1)	9.2 (4.6, 13.7)	3.3 (0.5, 6.1)	20.3 (13.9, 26.6)	17.0 (11.0, 22.9)
^a^ *P* value	>0.05	<0.01	>0.05	<0.05	<0.001	<0.001	<0.001	>0.05	<0.05

Data were presented as proportion and 95% confidence interval (95% CI). ^a^The Bonferroni correction was used to correct *P* values of pairwise comparison among five age groups.

**Figure 5:**
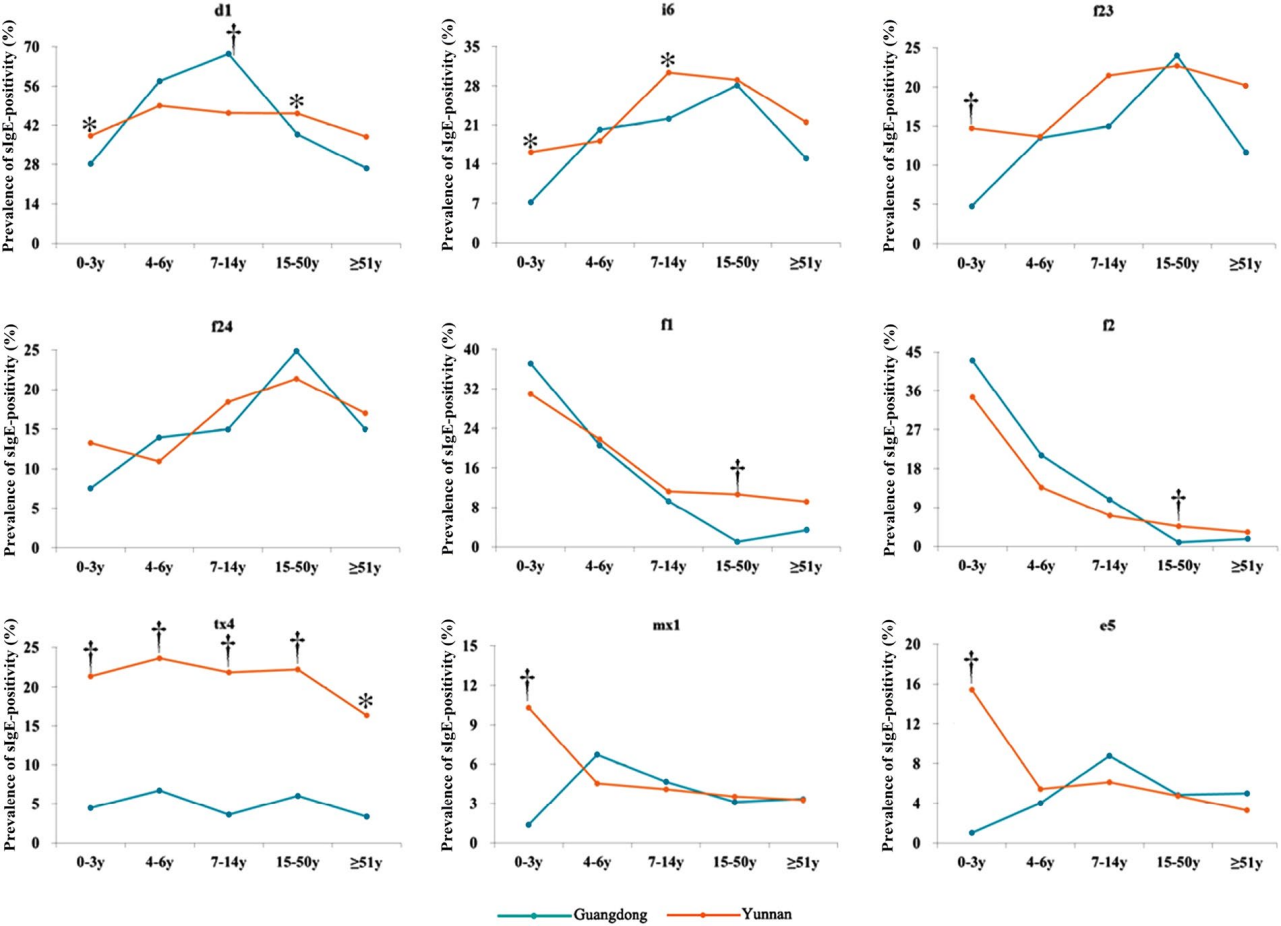
Distribution of allergen sIgE positivity among age groups in Guangdong and Yunnan. ^*^ *P* < 0.05, ^†^ *P* < 0.001. d1: house dust mite; i6: cockroach; tx4: tree pollen mix; mx1: mold mix; e5: dog dander; f23: crab; f24: shrimp; f1: egg white; f2: milk.

Regardless of no statistical difference among age groups in prevalence of sIgE positivity for tree pollen mix within either region, the values in Yunnan were higher than those in Guangdong for any given age group (all *P* < 0.001). The prevalence for dog dander-sIgE positivity among children aged 0–3 years in Yunnan was higher than that in Guangdong [15.4% (95% CI: 14.1%, 16.7%) vs 1.0% (95% CI: 0.1%, 1.4%), *X* ^*2*^ = 36.282, *P* < 0.001]. Interestingly, the prevalence of dog dander sensitization in Guangdong subjects aged 0–3 years was significantly lower compared with those aged 7–50 years (all *P* < 0.001); but in Yunnan, the opposite was noted. The same observation was obtained regarding the mold mix-sIgE positivity in the age group 0–3 years when compared between the two regions [10.3% (95% CI: 9.2%, 11.4%) vs 1.4% (95% CI: 0.1%, 1.8%), *X* ^*2*^ = 18.262, *P* < 0.001], or compared with those aged 7–50 years in the either region (Guangdong; *X* ^*2*^ = 11.420, *P* < 0.05: Yunnan; *X* ^*2*^ = 10.301, *P* < 0.05) (Fig. 5).

### Sensitization to house dust mite in relation to sIgE positivity for other inhalant/food allergens

**Figure 6:**
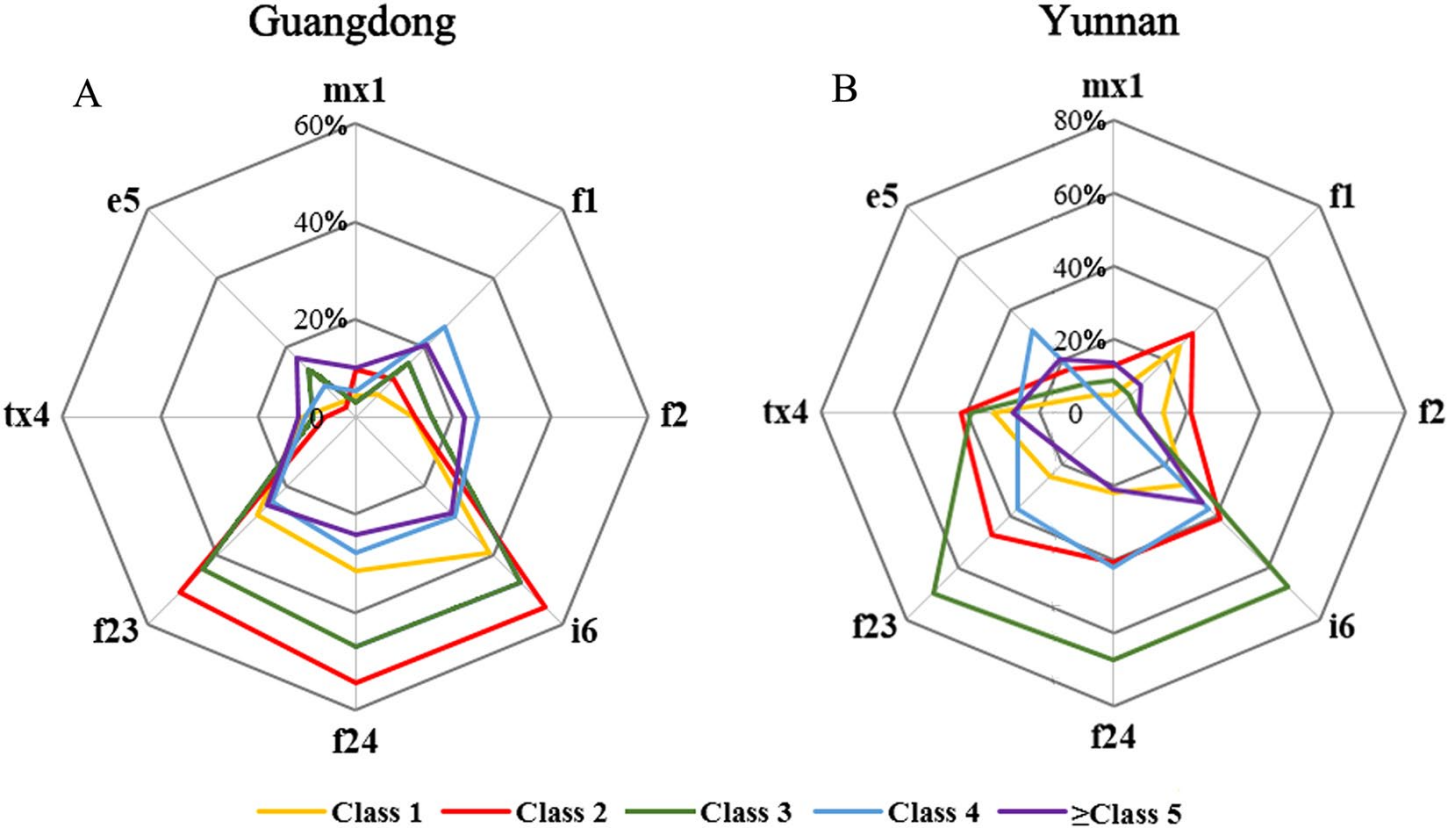
Radar charts of co-sensitization to other allergens in house dust mites (d1)-positive subjects from Guangdong (**A**, N = 583) and Yunnan (**B**, N = 642) (%). The classes in the figure indicate the d1 sIgE reactivity in house dust mite positive subjects. Plots more closer to the outer edge indicate that the d1-positive subjects of that class were more likely to be positive for a given allergen; plots more closer to the center indicate that the d1-positive subjects were less likely to be sensitized to the allergen. The subjects with d1 sIgE reactivity of Classes 2–3 were mostly co-sensitized to f23, f24, and i6 in the two regions. d1: house dust mite; i6: cockroach; tx4: tree pollen mix; mx1: mold mix; e5: dog dander; f23: crab; f24: shrimp; f1: egg white; f2: milk.

In light of house dust mite (d1) sensitization being predominant in both regions and presenting more high-class (≥4) reactivity in Guangdong, a specific sub-analysis using the radar chart was performed to explore the relationship in sensitization between house dust mite and other allergens. Among house dust mite-sensitized subjects in Guangdong and Yunnan, only 35.7% (208/583) and 22.9% (147/642), respectively, tested positive for house dust mite alone. Moreover, house dust mite-positive subjects were more likely to be positive to at least one of the other eight allergens, compared with those who were house dust mite-negative. Among these poly-sensitized subjects, cockroach was the most common co-sensitizing aeroallergen in both Guangdong (40.1%) and Yunnan subjects (36.4%). In Yunnan, a considerably high proportion of house dust mite-sensitized subjects (36.3%) were concomitantly positive for tree pollen mix. Co-sensitizing food allergens in house dust mite-positive subjects were mostly shrimp (Guangdong 37.6%; Yunnan 33.2%) and crab (Guangdong 35.3%; Yunnan 36.9%). Noticeably, the d1 sIgE reactivity was mainly classes 1–3 in poly-sensitized subjects (Fig. 6).

## Discussion

Investigational attempts to look at trans-regional difference in allergen sensitization within a vast geographical territory should be worthwhile to guide evidence-based prevention and treatment of local allergies. In this study, we examined the local common allergens affecting subjects with suspected atopy in the southeast coastal and southwest inland regions of China by measuring sIgE to nine inhalant/food allergens using a uniform implementation protocol for serum logistics and detection.

Overall, our findings revealed slightly higher prevalence of sIgE positivity for any allergen among males compared with females in both regions (*P* < 0.05). Such a disparity between genders may not reflect the natural occurrence in the general population, because the present study focused on subjects with suspected atopy. In a large sample study on 3371 patients with allergic rhinitis and/or asthma^18^, Boulet *et al*. found that male patients had a higher atopic index than did female patients, suggesting that within this patient subset, men are more susceptible to allergen sensitization than women. The present study also found that majority of sIgE-positive subjects were sensitized to two or more of the tested allergens (Guangdong: 56.5%; Yunnan: 69.3%). This should prompt for screening multiple allergens when evaluating an individual with suspected allergy. As far as sensitization to any allergen was concerned, the prevalence of sIgE positivity seemed to increase along with age, peaking either at 7–14 years (Guangdong: 75.3%) or at 15–50 years (Yunnan: 63.3%), and decreasing thereafter. That allergen sIgE-positive prevalence peaked during young adulthood in life has been documented in our previous studies^13^ and by others^18^. In contrast, declining of innate or adaptive immune functions known as “immunosenescence” in the elderly may result in weaker immune reactivity^19^, and hence the lower rate of any allergen sensitization in subjects aged ≥51 years compared with other age groups as we have noted in this study. However, prevalence of sIgE positivity to any allergen in Yunnan appeared to show milder age-group variation than those in Guangdong, and were significantly higher compared with Guangdong subjects for the age groups 15–50 years and ≥51 years (*P* < 0.05). We speculated that this might have been associated with the perennial presence of airborne pollen throughout the Yunnan province resulting in increased exposure and sensitization for the local population of different age groups^20^.

In the majority of south Asian regions, climates are typically subtropical, with warm and humid environments conducive to survival of dust mites as major aeroallergens^21–24^. In this study conducted in southern China, house dust mite (d1) was the predominant aeroallergen in both coastal and inland regions. It would be important to note that, unlike Yunnan where d1-sIgE reactivity was chiefly low (45.2% with class 1, 40.2% with class 2), 41.9% of d1-sIgE positive Guangdong subjects showed high-level responses (≥class 4). The high-class reactivity to house dust mite response in Guangdong echoed the findings by Wang *et al*.^25^ that derived from 215,210 cases of allergen sIgE detection. Despite the similar latitudinal range within which the both regions are located, the more frequent use of air-conditioning and hence inadequately ventilated indoor activities during summer in economically developed Guangdong might have added to severity of house dust mite sensitization, as has been documented^26, 27^. It would also be important to note that in either region, cockroach ranked the second in leading allergens, and that among poly-sensitized subjects with d1-positivity, cockroach was the most common co-sensitizing aeroallergen, although the proportion of sIgE positivity for cockroach was significantly higher in Yunnan than in Guangdong (*P* = 0.001). These observations could be supported by similarity in breeding environments between cockroach and house dust mite^28^, highly probable cross-reactivity between these two allergens^29^, and previous findings on higher sensitization rates for cockroaches in mountainous areas^30^.

The Yunnan province is widely known as a giant “botanical garden” of China, with copious resources of vegetation. A study in the region identified dispersion of airborne pollens all year round that culminates during the February to April and the September to November seasons^20^. Considerably, there could be a higher level of pollen exposure for Yunnan subjects compared to those in Guangdong, which may partly explained for the drastically higher sIgE positive rate for tree pollen mix (tx4) being four times as found for Guangdong (*P* < 0.001), and that up to 36.3% of house dust mite-positive Yunnan subjects were co-sensitized to tx4. However, the level of pollen grains in the atmosphere can be influenced by a number of meteorological and geomorphological factors. In a study, Bartková-Ščevkova found atmospheric pollen levels to be negatively correlated with relative humidity and rainfall in Bratislava of Slovakia^31^. The author, along with others^32^, pointed out that the negative correlation with rainfall would be more significantly when the rainfall within a longer period (days or months) or intense rainfall in a certain area is taken into consideration. Atmospheric pressure has also been indicated to be a factor affecting concentrations of pollen in the air^33^. A negative correlation between atmospheric pressure and airborne pollen grains was documented in a 3-year observational study by Stennett and Beggs in Sydney^34^. During the our study period between 2014 and 2015, the annual rainfall and relative humidity was 1078 millimeter and 67.6% in Yunnan, compared with 2234 millimeter and 77.8% in Guangdong^35^. In addition, Yunnan is geographically located on an average altitude of 1800–2000 meters vs 80–100 meters above sea level in most of Guangdong. Such a contrast in altitude may be translated into 20-kPa difference between the two regions, given an increase of 9 meters in height corresponding to a decrease of 100 Pascals in atmospheric pressure. These data could further explain the higher prevalence of tx4 sIgE positivity in Yunnan. Nevertheless, we believed that the trans-regional difference in sensitization to tx4 or any pollen between the both regions should inspire future studies rather than simply looking at the proportion of sIgE positivity. In fact, none of these single correlations may work individually in nature, and can be further complicated by plant diversity and meteorological heterogeneity between regions. Furthermore, we could not exactly explain for absence of aging effect in tree pollen mix sensitization (declining with age, as found for other allergens), since this was merely a cross-sectional observational study in a convenience sample of subjects with suspected atopy but not the general population. However, our findings could be practically useful and relevant for clinical settings.

Interesting data as regards to food allergens from Yunnan and Guangdong also appeared implicative. In this study, the major allergens for food sensitization in children aged 0–6 years were eggs and milk in both regions, and the prevalence of sIgE positivity for both allergens exhibited a decreasing trend later in life. This seemed to be a commonplace as it has been reported in a number of studies^13, 36–40^, but such a similarity as observed between Yunnan and Guangdong reiterated the importance of detection for egg and milk allergens among infants, toddlers and young children. We also noted a significantly higher prevalence of milk sIgE positivity in Guangdong for children aged 14 or below than for those in Yunnan. The better socioeconomic status and rapid westernization in lifestyle in the coastal Guangdong province may have contributed to frequent and more intake of milk, leading to a relatively higher rate of milk positivity among children in this region^13, 26^. As for seafood, consumption of shrimps, crabs, and other crustaceans is more usual in coastal than in inland regions. Surprisingly, we demonstrated that Yunnan subjects showed high prevalence of sIgE positivity for crabs and shrimps, and the sensitization to crab in Yunnan subjects of all age groups was even more prevalent than in their Guangdong counterparts (*P* < 0.05). Similar findings were demonstrated in a study on food sensitization by Yang *et al*.^30^ where they unexpectedly found higher prevalence of seafood sensitization in school-age children living in inland rural areas compared with those living in coastal cities (42.1% vs 25.9%, *P* < 0.05). We speculated that three aspects might be attributable to such a paradox. Firstly, sensitization to a certain allergen may arise from cross-reactions by tropomyosin, and these cross-reactions can be frustratingly common. Tropomyosin is known to be an allergen component found in house dust mite, cockroach, crab, and shrimp^15, 41^, and may be responsible for cross-reactions to cockroach, crab, and shrimp in house dust mite-positive subjects, leading to false-positive results and hence higher prevalence of sensitization. Second, the rate of sensitization to tropomyosin as an allergen component (Der p 10) of house dust mite can vary under different climates, ranging from 4% in subtropical to 8.9% in temperate regions^16, 42^. In our study, the higher positive rate of house dust mite in temperate Yunnan than in subtropical Guangdong (44.9% vs 43.3%) might raise the possibility of stronger tropomyosin cross-reactivity among Yunnan subjects, leading to higher prevalence of crab and shrimp sIgE positivity. Third, residents in Guangdong, particularly those from coastal regions, are more likely and frequently to consume shellfish such as shrimp and crab. For children, they may be exposed to seafood earlier in life. We speculated that, the continuous exposure might give rise to immune tolerance against the allergens of shellfish, which could reduce the risk of sensitization. This can be similar to the findings that children or adults living in rural areas or on farms are more likely to be exposed to various allergens but have lower incidence of allergic diseases than those living in cities^43^, although the exactly underlying mechanism needs further clarification.

Certain limitations were noted in the present study. Only nine common allergens were examined and compared between Yunnan and Guangdong, owing to lack of data on a full-spectrum of common allergens in the two regions, and inadequate commercially availability of standardized crude extract products. Thereby, other potentially common allergens in the two regions might have been missed. In addition, to facilitate obtaining a large number of serum samples in an ethically acceptable manner, the present study was designed to focus on a convenience sample of subjects with suspected atopy. Despite this, we speculated that a convenience sample would be more practically feasible for a preliminary and pioneering study, rendering future validation warranted. Furthermore, we did not examine the sIgE positivity with stratification by subtypes of allergic diseases because the number of subjects with each subtype varied widely and might not be powered for a sensible analysis. Notwithstanding these, research comparing the distribution of allergens across different regions and climates plays an important role in treatment of allergic diseases. The present study may offer valuable implications for local physicians in their clinical practice. Finally yet importantly, our findings would inspire comparative studies worldwide on allergen spectra across regions within similar latitudinal range but with distinct geography and climates.

In summary, we found a high prevalence of overall sIgE positivity to local allergens among subjects with suspected atopy in two southern China provinces about 840 miles apart. House dust mite (d1) was the most common sensitizing allergen in the two population subsets. Sensitization to d1 was frequently accompanied by co-sensitization to other local allergens (such as cockroach) in both regions and tree pollens in Yunnan. Among subjects in the inland Yunnan, sensitization to crab and shrimp were more common compared with those from the coastal Guangdong. These differences might be associated with differences in lifestyle, climates and geomorphological features between the two regions, although general population-based studies are lacking. While further validation and interpretation are needed, our findings may add to data for evidence-based management of local allergies in China and worldwide.
